# Supplementary material for: Phase diagrams of pharmaceutical solvates from mechanochemistry
Source: Nat Commun. 2026 Jan 7;17:1048. doi: 10.1038/s41467-025-67806-1 (PMC12847708; doi:10.1038/s41467-025-67806-1)
Supplement: Supplementary file 1 — Supplementary Information [file 41467_2025_67806_MOESM1_ESM.pdf]

Supplementary Information

Phase Diagrams of Pharmaceutical Solvates from Mechanochemistry

Fragkoulis Theodosiou[1], Toby J. Blundell[1], John S.O. Evans,[1] Patricia Basford[2,3], Noalle Fellah[4] and Aurora J. Cruz Cabeza[1*]

[1]Department of Chemistry, Durham University, Durham, DH1 3LE, UK
[2]Material Sciences, Pharmaceutical R&D, Pfizer Central Research, Kent, CT13 9NJ, UK
[3]Particology Ltd., Sandwich, Kent, CT13 9ND, UK
[4]Chemical Research and Development, Pfizer Inc., Groton Connecticut, CT 06340, USA
*email: aurora.j.cruz-cabeza@durham.ac.uk

1. Methods Supplementary Information 2
1.1 Materials 2
1.2 CSA-LAG Milling Experiments 2
1.3 Solubility Measurements 2
1.4 Solvent Mediated Phase Transformation (Slurries) 2
1.5 Solvent Activity Calculations 3
1.6 Binary Solvent Activity Mixtures 4
1.7 Ternary Solvent Activity Mixtures 6
1.8 Characterisation Techniques 7
1.8.1 Powder X-Ray Diffraction (PXRD) 7
1.8.2 Single Crystal X-Ray Diffraction (SCXRD) 9
1.8.3 Rietveld Refinement 9
2. Results Supplementary Information 11
2.1 Form Identification 11
2.2 Slurry Experiments 11
2.3 CSA-LAG Experiments with Binary Liquid Mixtures 14
2.4 Effective temperature working window under CSA-LAG 19
2.5 CSA-LAG Experiments of Competing Solvates with Binary Liquid Mixtures 20
2.6 CSA-LAG Experiments of Competing Solvates with Ternary Liquid Mixtures 20
2.7 Investigating Critical Water Activity Transferability 22
2.8 Crystal structures determined by SCXRD 23
Supplementary References 25

1. Methods Supplementary Information

1.1 Materials

Nitrofurantoin anhydrous (purity > 99%), theophylline anhydrous (purity > 99%), carbamazepine anhydrous (purity > 99%) and 4-hydroxybenzamide anhydrous (purity > 98%) were all procured from Sigma-Aldrich at the highest available purities. The material forms were identified by PXRD and were subsequently used without any further processing. Dry organic solvents including acetone (ace), N,N-Dimethylformamide (dmf), ethanol (etoh), isopropanol (ipa), and ethylene glycol (eg) of HPLC quality were obtained from Sigma and were used without further purification. Demineralised ultrapure water (h2o) was purchased from Fisher Scientific.

1.2 CSA-LAG Milling Experiments

CSA-LAG experiments were conducted using a Retsch MM400 Mixer Mill with screw top 10ml zirconium oxide milling jars and a single 10 mm zirconium oxide ball, fitted with a Teflon gasket to ensure an environmental seal. 100 mg of solids and 200 µL of (CSA) solutions were used. After preparation of the jars, they were precooled in the fridge until their temperature reached 20 °C (as measured with an infrared thermometer). At that point, the jars were loaded onto the MM400 and the samples milled at a frequency of 30 Hz for 30 minutes. Immediately after milling, the jars were removed and the temperature monitored on the jar surface using an infrared thermometer. In all experiments, the recorded temperature ranged from 24 to 26 °C.

1.3 Solubility Measurements

Solubility measurements at variable conditions for hydrate/anhydrate systems in solutions of controlled solvent activity were determined via gravimetric methods. Equilibration was achieved whilst stirring excess solids in solutions under constant temperature 25ºC for 1 week. The suspended crystals were allowed to settle over 24h, and at each condition, aliquots were collected and let to evaporate in pre-weighted vials. Samples of three were collected via gravimetric approach for each case studied via gravimetric solubility method.

1.4 Solvent Mediated Phase Transformation (Slurries)

For the determination of the critical water/solvent activities at a steady state defined temperature and pressure, the method of controlled solvent activity slurry bridging was implemented. The prepared solvent mixtures of desired activities were saturated with the anhydrous form of the API of interest. Next, the solutions were supersaturated by the addition a 50:50 mixture of the two competing forms (i.e. hydrate and anhydrous), to allow the bridging of the two forms bypassing potential nucleation limitations. The resulting suspensions were sealed with PTFE screw top lids, tightly wrapped with several parafilm layers to limit any potential solvent permeation. Using magnetic stirrer bars, the solutions were agitated at a constant rate and temperature (25 °C) using the Polar Bear Plus Crystal (Cambridge Reactor Design, Cambridge, United Kingdom). After the duration of two weeks, the weight of the vials measured to validate no significant solvent loss, and the resulting precipitated crystals were analysed via PXRD.

1.5 Solvent Activity Calculations

For the calculation of the desired solvent activities for binary and tertiary solvent mixtures targeting the formation of hydrates, solvates, or anhydrous phases, the non-random-two-liquid model (NRTL) was used and implemented from ASPEN Plus v14.1[1]. The NRTL model was selected as it is applicable for ambient environmental conditions, complete miscibility of components and multi-component interactions[2,3]. The model parameters are defined from fitted experimental data of steady-state vapour liquid equilibria (VLE), available in the NIST, ASPEN, DECHEMA and IG databases. Alternatively, other calculation approaches include the Wilson Theory, (UNIversal QUAsi Chemical model) UNIQUAC and (UNIversal Function Activity Coefficient Model) UNIFAC, all of which are commonly used by engineers to model stream properties in the chemical and pharmaceutical industries[2,4]. Since solvent-solvent interactions dominate the solvent activities and the NRTL derived activities provide a valid approximation, as also applied in slurry-based studies[5–9] and industrial crystallisation practice. Any local perturbations induced by solute-solvent interactions would only slightly shift the observed thresholds, which are implicitly accounted for in the experimental phase boundaries reported.

For any multicomponent mixture, at steady state, the chemical potential (μ_i) of each component is in phase equilibrium resulting in Eq 1. This can be redefined as Eq 2, where the activity of the compound (α_i) is linked with its molarity (x_i), the unit activity (μ_i^*) and the activity coefficient (γ_i). The absence of ideal mixing introduces the need for the presence of the activity coefficient (γ) which rectifies the non-ideality of mixing arising from the variable intermolecular interactions of different molecules and poses a crucial role in the modelling of mixture properties, such as solvent and water activities in this context. The activity of a species (α_i) is determined by Eq 3, as a result of the product of the molarity and activity coefficient[4,7,8,10–12]. In the case that solvent is water, the relative humidity would be the percentile water activity, ie Eq 4.

$$\mu_i^V = \mu_i^L \quad (1)$$

$$\mu_i^* + RTln(\alpha_i) = \mu_i^* + RTln(x_i) + RTln(\gamma_i) \quad (2)$$

$$\alpha_i = \gamma_i \times x_i \quad (3)$$

$$RH = \alpha_i \times 100\% \quad (4)$$

The solvent selection must ensure complete miscibility of the components of the mixtures in all ratios allowing for a uniform activity throughout the liquid phase and the liquid-vapor phase. All liquid volume handling was performed using Eppendorf Research Plus micropipettes and single use tips for all decanting (Eppendorf, Hamburg, Germany). The resulting mixtures were vortex-mixed for one minute after preparation and every time prior to milling to ensure homogeneous mixing and thus activity uniformity throughout the solution. Mixtures were typically prepared on a 10 ml scale to account for evaporative losses, stored in screw top vials with polyvinyl faced liners and tightly wrapped with parafilm along the cap and glass seal to minimise solvent losses. Weight monitoring of the vial is suggested after long term storage.

1.6 Binary Solvent Activity Mixtures

Tables S1 to S6 below give details for the preparation of the various binary solvent mixtures used in this work and the calculated activities for all components.

Table S 1. Water:Ethanol mixtures: Volume and mol fraction of h2o used to prepare h2o:ethoh mixtures and the resulting solvent activities.

h2o Volume Fraction	h2o Mol Fraction	α_{h2o}	α_{etoh}
0.000	0.000	0.000	1.000
0.013	0.041	0.103	0.960
0.028	0.085	0.203	0.920
0.046	0.134	0.302	0.877
0.069	0.193	0.402	0.829
0.082	0.224	0.448	0.806
0.100	0.264	0.503	0.777
0.119	0.303	0.550	0.750
0.144	0.352	0.601	0.718
0.220	0.476	0.702	0.644
0.380	0.664	0.802	0.541
0.673	0.869	0.900	0.355
1.000	1.000	1.000	0.000

Table S 2. Water:acetone mixtures: Volume and mol fraction of h2o used to prepare h2o:ace mixtures and the resulting solvent activities.

h2o Volume Fraction	h2o Mol Fraction	α_{h2o}	α_{ace}
0.000	0.000	0.000	1.000
0.008	0.032	0.108	0.969
0.016	0.062	0.198	0.941
0.027	0.102	0.301	0.907
0.041	0.149	0.403	0.871
0.059	0.204	0.500	0.831
0.086	0.278	0.600	0.785
0.134	0.388	0.700	0.728
0.263	0.594	0.800	0.642
0.605	0.863	0.900	0.442
1.000	1.000	1.000	0.000

Table S 3. Water:ethylene glycol mixtures: Volume and mol fraction of h2o used to prepare h2o:eg mixtures and the resulting solvent activities.

h2o Volume Fraction	h2o Mol Fraction	α_{h2o}	α_{eg}
0.000	0.000	0.000	1.000
0.043	0.122	0.108	0.876
0.083	0.219	0.198	0.776
0.134	0.324	0.301	0.667
0.194	0.427	0.403	0.559
0.223	0.471	0.448	0.513
0.260	0.521	0.500	0.460
0.299	0.569	0.550	0.411
0.342	0.617	0.600	0.361
0.444	0.712	0.700	0.266
0.574	0.807	0.800	0.173
0.748	0.902	0.900	0.085
1.000	1.000	1.000	0.000

Table S 4. DMSO:Ethanol mixtures: Volume and mol fraction of dmso used to prepare dmso:etoh mixtures and the resulting solvent activities.

dmso Volume Fraction	dmso Mol Fraction	α_{dmso}	α_{etoh}
0.000	0.000	0.000	1.000
0.251	0.215	0.100	0.717
0.374	0.328	0.200	0.556
0.47	0.420	0.300	0.437
0.553	0.502	0.400	0.341
0.629	0.580	0.500	0.263
0.702	0.658	0.600	0.195
0.774	0.736	0.700	0.137
0.846	0.818	0.799	0.086
0.921	0.905	0.900	0.040
1.000	1.000	1.000	0.000

Table S 5. DMF:Ethanol mixtures: Volume and mol fraction of dmf used to prepare dmf:etoh mixtures and the resulting solvent activities.

dmf Volume Fraction	dmf Mol Fraction	α_{dmf}	α_{etoh}
0.000	0.000	0.000	1.000
0.252	0.202	0.100	0.754
0.388	0.323	0.200	0.591
0.491	0.420	0.301	0.465
0.577	0.506	0.401	0.363
0.653	0.586	0.500	0.278
0.725	0.665	0.601	0.205
0.793	0.742	0.701	0.142
0.861	0.823	0.802	0.087
0.921	0.905	0.900	0.041
1.000	1.000	1.000	0.000

Table S 6. Acetone:Ethylene glycol mixtures: Volume and mol fraction of ace used to prepare ace:eg mixtures and the resulting solvent activities.

ace Volume Fraction	ace Mol Fraction	α_{ace}	α_{eg}
0.000	0.000	0.000	1
0.027	0.021	0.103	0.980
0.056	0.043	0.200	0.960
0.090	0.070	0.299	0.938
0.131	0.102	0.400	0.913
0.181	0.143	0.500	0.885
0.248	0.199	0.600	0.853
0.349	0.288	0.700	0.812
0.580	0.511	0.800	0.749
0.893	0.863	0.900	0.526
1.000	1.000	1.000	0

1.7 Ternary Solvent Activity Mixtures

Table S7 below gives details for the preparation of the dmf:ethanol:water ternary liquid mixtures and the calculated activities for all components. Parameters for the estimation of the gamma coefficients for the components were extracted from the NIST and ASPEN databases.

Table S 7. DMF:water:ethanol mixture ternary molar composition of dmf, h2o and etoh used to prepare mixtures of known solvent activities.

Set	x_{h2o}	x_{etoh}	x_{dmf}	α_{h2o}	α_{etoh}	α_{dmf}
1	0.7863	0.1220	0.0917	0.8234	0.2645	0.1056
2	0.7340	0.1518	0.1141	0.7853	0.3167	0.0554
3	0.6479	0.2010	0.1511	0.7225	0.3533	0.0741
4	0.4791	0.2973	0.2235	0.5920	0.3989	0.1151
5	0.1554	0.4822	0.3624	0.2570	0.4436	0.2251
6	0.0842	0.5228	0.3930	0.1519	0.4490	0.2601
7	0.0440	0.5458	0.4102	0.0838	0.4517	0.2828
8	0.0913	0.0567	0.8520	0.0701	0.0280	0.8384
9	0.0733	0.9096	0.0171	0.1818	0.9151	0.0083
10	0.7008	0.2175	0.0817	0.7824	0.4120	0.0348
11	0.6024	0.1869	0.2107	0.6663	0.3031	0.1158
12	0.4975	0.1544	0.3481	0.5266	0.2002	0.2395
13	0.3690	0.1145	0.5164	0.3559	0.1087	0.4305
14	0.2808	0.5227	0.1965	0.4596	0.5753	0.0894
15	0.2347	0.4369	0.3284	0.3563	0.4359	0.1922
16	0.2016	0.3753	0.4231	0.2810	0.3385	0.2901
17	0.1697	0.3159	0.5145	0.2142	0.2541	0.3993
18	0.1465	0.2727	0.5808	0.1707	0.2003	0.4845
19	0.1418	0.6598	0.1984	0.2998	0.6634	0.0903
20	0.1252	0.5828	0.2920	0.2440	0.5534	0.1605
21	0.0668	0.6217	0.3115	0.1443	0.5646	0.1812
22	0.0791	0.7364	0.1845	0.1999	0.7228	0.0828
23	0.4385	0.4081	0.1534	0.6016	0.5234	0.0667
24	0.3355	0.3123	0.3522	0.4269	0.3344	0.2210
25	0.3003	0.2795	0.4202	0.3646	0.2740	0.2933
26	0.2717	0.2529	0.4753	0.3156	0.2292	0.3576
27	0.0278	0.6476	0.3245	0.0648	0.5716	0.1976
28	0.0303	0.7048	0.2649	0.0785	0.6495	0.1445
29	0.0346	0.8042	0.1612	0.1054	0.7836	0.0704
30	0.4238	0.1315	0.4448	0.4271	0.1430	0.3453
31	0.5953	0.0924	0.3124	0.5983	0.1426	0.2194
32	0.6645	0.1031	0.2325	0.6843	0.1872	0.1456
33	0.0368	0.8560	0.1072	0.1183	0.8492	0.0413
34	0.5500	0.3500	0.1000	0.6945	0.5111	0.0408
35	0.4000	0.5000	0.1000	0.5960	0.6092	0.0399
36	0.3000	0.6000	0.1000	0.5098	0.6724	0.0399
37	0.2000	0.7000	0.1000	0.3992	0.7385	0.0395

1.8 Characterisation Techniques

1.8.1 Powder X-Ray Diffraction (PXRD)

PXRD patterns of the milled and slurried samples were all acquired on a Brucker AXS D8 Advance CX004310 diffractometer fitted with a Lynxeye Soller PSD Detector, in Bragg-Brentano geometry using Cu Ka radiation (λ=1.5406 Å) and a Ni filter with the variable slits set at 6 mm. The Bragg reflections were collected on a 2θ range of 2-50 at a time step of 0.5 and a 2θ increment of 0.02° per step.

Samples were analysed on Si zero background wafer packs with material wells as seen in Figure S1. To minimise environmental effects and to maintain the steady-state solvent activity achieved in the mill, approximately 20 mg of material was placed in the Si well and was immediately covered with 0.01 mm Kapton (polyamide) tape. All samples were prepared and analysed immediately after the opening of the milling jars, as to preserve the steady state activity environment reached through the milling process. The scattering contributions of the Kapton tape were collected and background subtracted from the diffracted patterns.

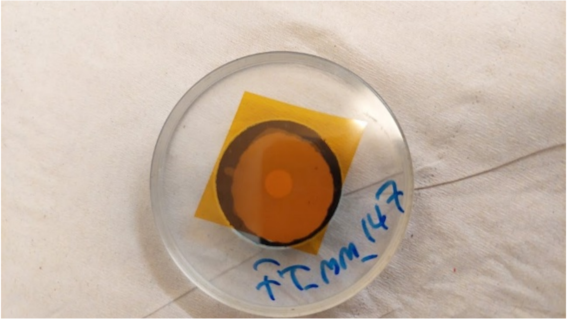

Figure S 1: PXRD sample pack with Kapton Tape cover.

An example of the raw collected background data is presented below in Figure S2 in black. As it can be observed the scattering contributions arising from the use of Kapton are depicted by the red pattern. In addition the increased background intensity at higher diffraction angles arises from the Bragg-Brentano geometry with a variable divergence slit, where the illuminated area of the sample grows with angle, thereby enhancing diffuse scattering contributions.

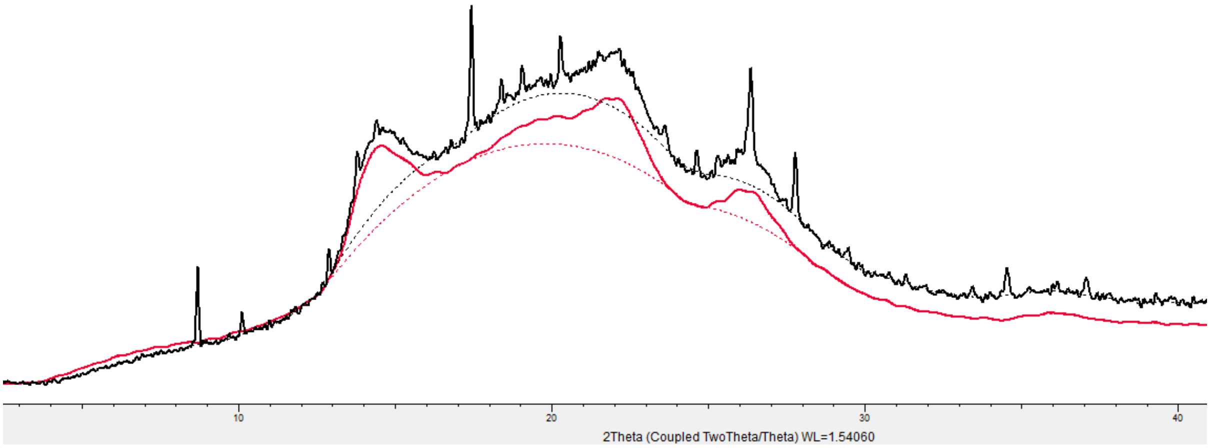

Figure S 2: Example of raw PXRD data (black) collected from a CSA-LAG sample covered with Kapton. The red pattern represents the diffuse scattering contributions from the Kapton layer and the sample holder.

By performing a simple PXRD pattern subtraction, available feature in DIFFRAC.EVA or TOPAS the Kapton contributions can easily be removed, thus yielding to clean diffraction patterns that can directly be used for form identification or Rietveld refinement. As shown in Figure S3, diffraction data collected from crystalline samples sealed under a Kapton polyamide layer (black) inevitably include contributions from the Kapton film. The corresponding broad scattering contributions of the Kapton film (X-ray transparent), depicted in red, are collected and subsequently subtracted to remove the artificial halo and artefacts introduced by the contributions and the scattering of the Kapton tape. The resulting diffraction pattern in blue represents the intrinsic diffraction of the crystalline material, which is completely ‘halo’ free and thus of good crystallinity.

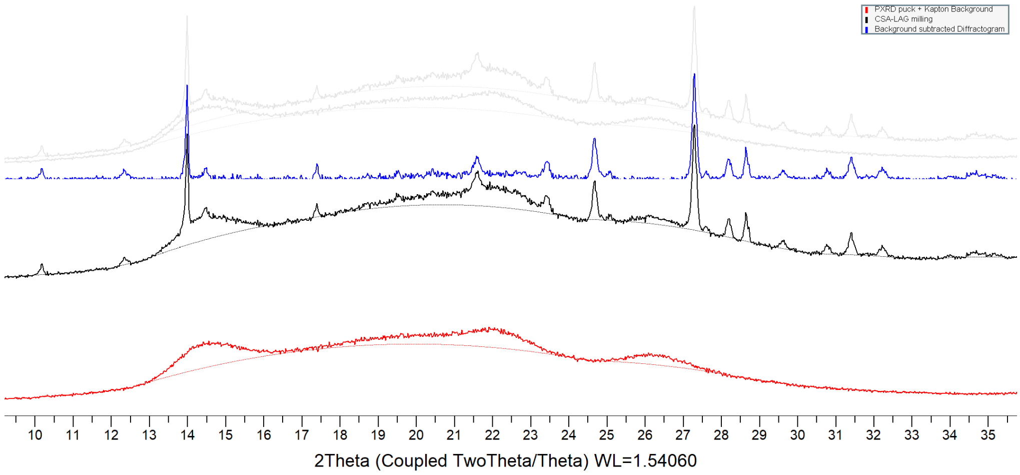

Figure S 3: Example of background subtraction using of PXRD patterns collected using an X-ray transparent film (Kapton). As it can be seen, the diffraction patters of Kapton (red) shows amorphous traits, with four characteristic “humps”. A sample PXRD pattern of a crystalline material produced under CSA-LAG conditions packed using a protective Kapton film (black). The “clean” Kapton subtracted powder pattern of the crystalline material (blue).

1.8.2 Single Crystal X-Ray Diffraction (SCXRD)

SCXRD was conducted for the structural elucidation of 4-hydroxybenzamide acetone solvate (4OHBZM-ACET-I) and nitrofurantoin dimethylformamide solvate (NF-DMF-II).

The X-ray single crystal data for 4OHBZM-ACET-I were collected at a temperature of 120.0(2) K using MoKα radiation (λ = 0.71073Å) on a Bruker D8 Venture with a Photon III MM C14 CPAD detector, IμS-III-microsource, focusing mirrors diffractometer equipped with a Cryostream (Oxford Cryosystems 700) open-flow nitrogen cryostat. The data for NF-DMF-II were collected at 100.0(2) K at the I-19 beamline (Dectris Pilatus 2M pixel-array photon-counting detector, undulator, graphite monochromator, λ = 0.68890 Å) at the Diamond Light Source, Oxfordshire and processed using Xia2/DIALS[13–17].

The structures were solved using Olex2[18] with the ShelXT[19] structure solution program using Intrinsic Phasing and refined with the ShelXL[20] refinement package using Least Squares minimization on F^2. All non-hydrogen atoms were refined with anisotropic displacement parameters. Hydrogen atoms were located in the difference map and refined isotropically using a riding model unless otherwise specified. Crystallographic data for 4OHBZM-ACET-I and NF-DMF-II have been deposited with the Cambridge Crystallographic Data Centre with deposition numbers CCDC-2443101 and CCDC-2443102 respectively.

1.8.3 Rietveld Refinement

PXRD patterns of the milled and slurried samples were all acquired on a Brucker AXS D8 Advance CX004310 diffractometer fitted with a Lynxeye Soller PSD Detector, in Bragg-Brentano geometry using a Cu Ka radiation (λ=1.5406 Å) and a Ni filter with the variable slits set at 6 mm. The Bragg reflections were collected on a 2θ range of 2-50 at a time step of 0.5 and a 2θ increment of 0.02° per step.

Quantitative analysis was performed using the Rietveld method using TOPAS ACADEMIC version 8[13,14,21,22]. Structural models for BAPLOT01 and THEOPH05 were taken from the CCDC and fractional coordinates fixed. A single overall isotropic temperature factor was refined for all atomic sites. The complex background due to the sample holder was modelled using 16 parameters. One parameter was used to scale a separately-measured scan of the empty holder to the data; an additional 9 parameters were used to describe Voigtian functions to fit broad Kapton arising background "humps"; and an additional 6 parameters described a smoothly-varying Chebychev polynomial. Preferred orientation was corrected using a March Dollase 1 parameter preferred orientation model. Rietveld-extracted weight percentages were 29.5(5)% and 70.5(5)% for BAPLOT01 and THEOPH05 respectively.

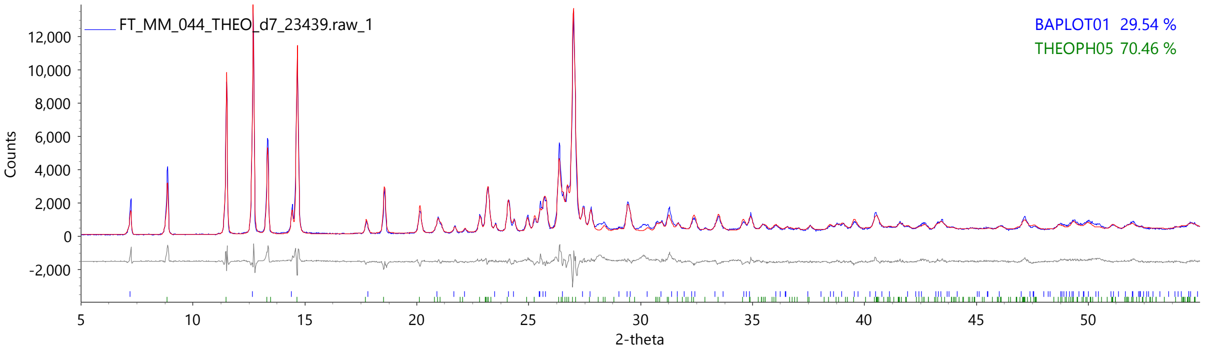

Figure S 4: Rietveld refitment of theophylline anhydrous milled at a water activity of 0.6, the resulting CSA-LAG pattern showing contributions from 29.5(5)% and 70.5(5)% for BAPLOT01 and THEOPH05. Observed data (blue) calculated fit (red) and difference profile (grey) are displayed.

Quantitative analysis was performed using the Rietveld method using TOPAS ACADEMIC version 8[13,14,21,22]. Structural models for LABJON02 (room temperature redetermination of LABLON) and 2443102 (NF-DMF-II) were taken from the CCDC and fractional coordinates fixed. A single overall isotropic temperature factor was refined for all atomic sites. Peak shapes were described using a Thomas-Cox-Hastings pseudo Voigt function with axial asymmetry and variable divergence intensity. The background was modelled with a 6 smoothly-varying Chebychev polynomial and an additional $1/x$ term to capture low angle scattering. Preferred orientation was corrected using 4th der spherical harmonics for each phase. Crystallite size and microstrain contributions were also refined. Rietveld-extracted weight percentages were 28.4(5)% and 71.5(5)% for 2443102 (NF-DMF-II) and LABJON02 respectively.

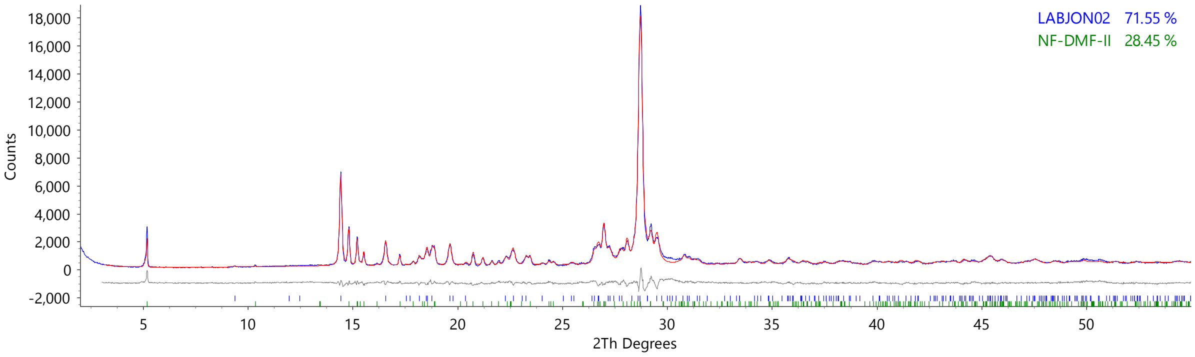

Figure S 5: Rietveld refitment of nitrofurantoin anhydrous milled at a dmf activity of 0.1, the resulting CSA-LAG pattern showing contributions from 28.4(5)% and 71.5(5)% for 2443102 (NF-DMF-II) and LABJON02 Observed data (blue) calculated fit (red) and difference profile (grey) are displayed.

2. Results Supplementary Information

2.1 Form Identification

Crystal structures for all four systems identified from crystallisation/milling/slurring products were identified by comparing the experimental PXRD with the simulated patterns from single crystal structures obtained from the Cambridge Structural Database (CSD). Systems, crystal forms, CSD refcodes, crystal compositions and liquid mixtures used for exploration are summarised in table S8 using the nomenclature adopted.

Table S8. Summary of the systems, crystal forms, CSD-refcodes, crystal form compositions and liquid mixtures used.

**System**	**Crystal Form**	**CSD-refcode**	**Crystal Composition**	**CSA-LAG/Slurry Liquid mixture (Active:**Carrier)[a]
theophylline (theo)	THEO-II<br>THEO-MH-I<br>THEO-DMSO-I	BAPLOT01<br>THEOPH05<br>RIGYEM	theo<br>theo:h_2o<br>theo:dmso	-<br>**h_2o**:ace<br>**dmso**:etoh
4-hydroxybenzamide (4ohbzm)	4OHBZM-I<br>4OHBZM-HH-I<br>4OHBZM-MH-I<br>4OHBZM-ACET-I	VIDMAX<br>GESCOY<br>JIXCOI01<br>2443101[b]	4ohbzm<br>4ohbzm:$(h_2o)_{0.5}$<br>4ohbzm: h_2o<br>4ohbzm:ace	-<br>**h_2o**:eg<br>**h_2o**:eg<br>**ace**:eg
carbamazepine (cbz)	CBZ-III<br>CBZ-DH-I<br>CBZ-DMSO-I	CBMZPN02<br>FEFNOT<br>UNEYIV01	cbz<br>cbz:$(h_2o)_2$<br>cbz:dmso	-<br>**h_2o**:etoh<br>**dmso**:etoh
nitrofurantoin (nf)	NF-β<br>NF-MH-II<br>NF-DMF-II	LABJON<br>HAXBUD<br>2443102[b]	nf<br>nf:h_2o<br>nf:dmf	-<br>**h_2o**:eg<br>**dmf**:etoh

[a] Liquid mixtures used for CSA-LAG in scenarios (a) and (b). Compositions were explored for mixtures with activities from 0 to 1 in steps of 0.1.[b] CSD deposition numbers for the new solvates identified in this work.

2.2 Slurry Experiments

Slurry experiments at 25°C were conducted to obtain critical solvent activities using the widely accepted competitive solution based approach. Results for all slurry experiments are presented below in tables S9 and S10 grouped as hydrates (S9) and other solvates (S10).

Table S 9: Solid form outcomes from slurry experiments at 25°C for 4ohbzm and nf. The active solvent is always **h2o**. The carrier solvents are eg and etoh. Slurries hydrate boundaries for theo and cbz were retrieved from the literature.

API	Slurry Liquid mixture **active:**carrier	α_{h2o}	Forms Identified
4ohbzm	**h2o**:eg	0.000	4OHBZM-I
4ohbzm	**h2o**:eg	0.108	4OHBZM-I
4ohbzm	**h2o**:eg	0.198	4OHBZM-I
4ohbzm	**h2o**:eg	0.301	4OHBZM-I
4ohbzm	**h2o**:eg	0.403	4OHBZM-I
4ohbzm	**h2o**:eg	0.500	4OHBZM-HH-I
4ohbzm	**h2o**:eg	0.600	4OHBZM-HH-I
4ohbzm	**h2o**:eg	0.700	4OHBZM-MH-I
4ohbzm	**h2o**:eg	0.800	4OHBZM-MH-I
4ohbzm	**h2o**:eg	0.900	4OHBZM-MH-I
4ohbzm	**h2o**:eg	1.000	4OHBZM-MH-I
nf	**h2o**:eg	0.000	NF-β
nf	**h2o**:eg	0.103	NF-β
nf	**h2o**:eg	0.203	NF-β
nf	**h2o**:eg	0.302	NF-β
nf	**h2o**:eg	0.400	NF-β
nf	**h2o**:eg	0.448	NF-β
nf	**h2o**:eg	0.550	NF-MH-I
nf	**h2o**:eg	0.601	NF-MH-I
nf	**h2o**:eg	0.702	NF-MH-I
nf	**h2o**:eg	0.802	NF-MH-I
nf	**h2o**:eg	0.900	NF-MH-I
nf	**h2o**:eg	1.000	NF-MH-I

Table S 10: Solid form outcomes from slurry experiments at 25°C for solvates exploration experiments for all four API systems. Active solvents are either dmso, ace or dmf. Carrier solvent used is always etoh.

API	Slurry Liquid mixture **active:**carrier	$\alpha_{active\ solvent}$	Forms Identified
theo	**dmso**:etoh	0.000	THEO-II
theo	**dmso**:etoh	0.100	THEO-II
theo	**dmso**:etoh	0.200	THEO-II
theo	**dmso**:etoh	0.300	THEO-DMSO-I
theo	**dmso**:etoh	0.400	THEO-DMSO-I
theo	**dmso**:etoh	0.500	THEO-DMSO-I
theo	**dmso**:etoh	0.600	THEO-DMSO-I
theo	**dmso**:etoh	0.700	THEO-DMSO-I
theo	**dmso**:etoh	0.799	THEO-DMSO-I
theo	**dmso**:etoh	0.900	THEO-DMSO-I
theo	**dmso**:etoh	1.000	THEO-DMSO-I
4ohbzm	**ace**:eg	0.000	4OHBZM-I
4ohbzm	**ace**:eg	0.100	4OHBZM-I
4ohbzm	**ace**:eg	0.200	4OHBZM-I
4ohbzm	**ace**:eg	0.300	4OHBZM-ACET-I
4ohbzm	**ace**:eg	0.401	4OHBZM-ACET-I
4ohbzm	**ace**:eg	0.508	4OHBZM-ACET-I
4ohbzm	**ace**:eg	0.600	4OHBZM-ACET-I
4ohbzm	**ace**:eg	0.700	4OHBZM-ACET-I
4ohbzm	**ace**:eg	0.799	4OHBZM-ACET-I
4ohbzm	**ace**:eg	0.901	4OHBZM-ACET-I
4ohbzm	**ace**:eg	1.000	4OHBZM-ACET-I
cbz	**dmso**:etoh	0.000	CBZ-III
cbz	**dmso**:etoh	0.100	CBZ-III
cbz	**dmso**:etoh	0.200	CBZ-III
cbz	**dmso**:etoh	0.300	CBZ-DMSO-I
cbz	**dmso**:etoh	0.400	CBZ-DMSO-I
cbz	**dmso**:etoh	0.500	CBZ-DMSO-I
cbz	**dmso**:etoh	0.600	CBZ-DMSO-I
cbz	**dmso**:etoh	0.700	CBZ-DMSO-I
cbz	**dmso**:etoh	0.799	CBZ-DMSO-I
cbz	**dmso**:etoh	0.900	CBZ-DMSO-I
cbz	**dmso**:etoh	1.000	CBZ-DMSO-I
nf	**dmf**:etoh	0.000	NF-β
nf	**dmf**:etoh	0.100	NF-DMF-II
nf	**dmf**:etoh	0.200	NF-DMF-II
nf	**dmf**:etoh	0.301	NF-DMF-II
nf	**dmf**:etoh	0.401	NF-DMF-II
nf	**dmf**:etoh	0.500	NF-DMF-II
nf	**dmf**:etoh	0.601	NF-DMF-II
nf	**dmf**:etoh	0.701	NF-DMF-II
nf	**dmf**:etoh	0.802	NF-DMF-II
nf	**dmf**:etoh	0.900	NF-DMF-II
nf	**dmf**:etoh	1.000	NF-DMF-II

2.3 CSA-LAG Experiments with Binary Liquid Mixtures

CSA-LAG experiments at 25±1°C were conducted to obtain critical solvent activities using our newly developed method. Results are presented below in tables S11 and S12 grouped as hydrates (S11) and other solvates (S12) with corresponding Figures S6 and S7.

Table S 11: Solid form outcomes from CSA-LAG experiments at 25±1°C for hydrates exploration experiments for all four API systems. The active solvent is always **h2o**. The carrier solvents are ace, eg and etoh.

API	CSA-LAG mixture (active:carrier)	α_{h2o}	Forms Identified
theo	**h2o**:ace	0.000	THEO-II
theo	**h2o**:ace	0.108	THEO-II
theo	**h2o**:ace	0.198	THEO-II
theo	**h2o**:ace	0.301	THEO-II
theo	**h2o**:ace	0.403	THEO-II
theo	**h2o**:ace	0.500	THEO-II
theo	**h2o**:ace	0.600	THEO-MH-I + THEO-II
theo	**h2o**:ace	0.700	THEO-MH-I
theo	**h2o**:ace	0.800	THEO-MH-I
theo	**h2o**:ace	0.900	THEO-MH-I
theo	**h2o**:ace	1.000	THEO-MH-I
4ohbzm	**h2o**:eg	0.000	4OHBZM-I
4ohbzm	**h2o**:eg	0.108	4OHBZM-I
4ohbzm	**h2o**:eg	0.198	4OHBZM-I
4ohbzm	**h2o**:eg	0.301	4OHBZM-I
4ohbzm	**h2o**:eg	0.403	4OHBZM-I
4ohbzm	**h2o**:eg	0.500	4OHBZM-HH-I
4ohbzm	**h2o**:eg	0.600	4OHBZM-HH-I
4ohbzm	**h2o**:eg	0.700	4OHBZM-HH-I
4ohbzm	**h2o**:eg	0.800	4OHBZM-MH-I
4ohbzm	**h2o**:eg	0.900	4OHBZM-MH-I
4ohbzm	**h2o**:eg	1.000	4OHBZM-MH-I
cbz	**h2o**:etoh	0.000	CBZ-III
cbz	**h2o**:etoh	0.103	CBZ-III
cbz	**h2o**:etoh	0.203	CBZ-III
cbz	**h2o**:etoh	0.302	CBZ-III
cbz	**h2o**:etoh	0.402	CBZ-III
cbz	**h2o**:etoh	0.503	CBZ-III
cbz	**h2o**:etoh	0.601	CBZ-III
cbz	**h2o**:etoh	0.702	CBZ-DH-I
cbz	**h2o**:etoh	0.802	CBZ-DH-I
cbz	**h2o**:etoh	0.900	CBZ-DH-I
cbz	**h2o**:etoh	1.000	CBZ-DH-I
nf	**h2o**:eg	0.000	NF-β
nf	**h2o**:eg	0.103	NF-β
nf	**h2o**:eg	0.203	NF-β
nf	**h2o**:eg	0.302	NF-β
nf	**h2o**:eg	0.400	NF-β
nf	**h2o**:eg	0.448	NF-β
nf	**h2o**:eg	0.550	NF-MH-I + NF-β
nf	**h2o**:eg	0.601	NF-MH-I
nf	**h2o**:eg	0.702	NF-MH-I
nf	**h2o**:eg	0.802	NF-MH-I
nf	**h2o**:eg	0.900	NF-MH-I
nf	**h2o**:eg	1.000	NF-MH-I

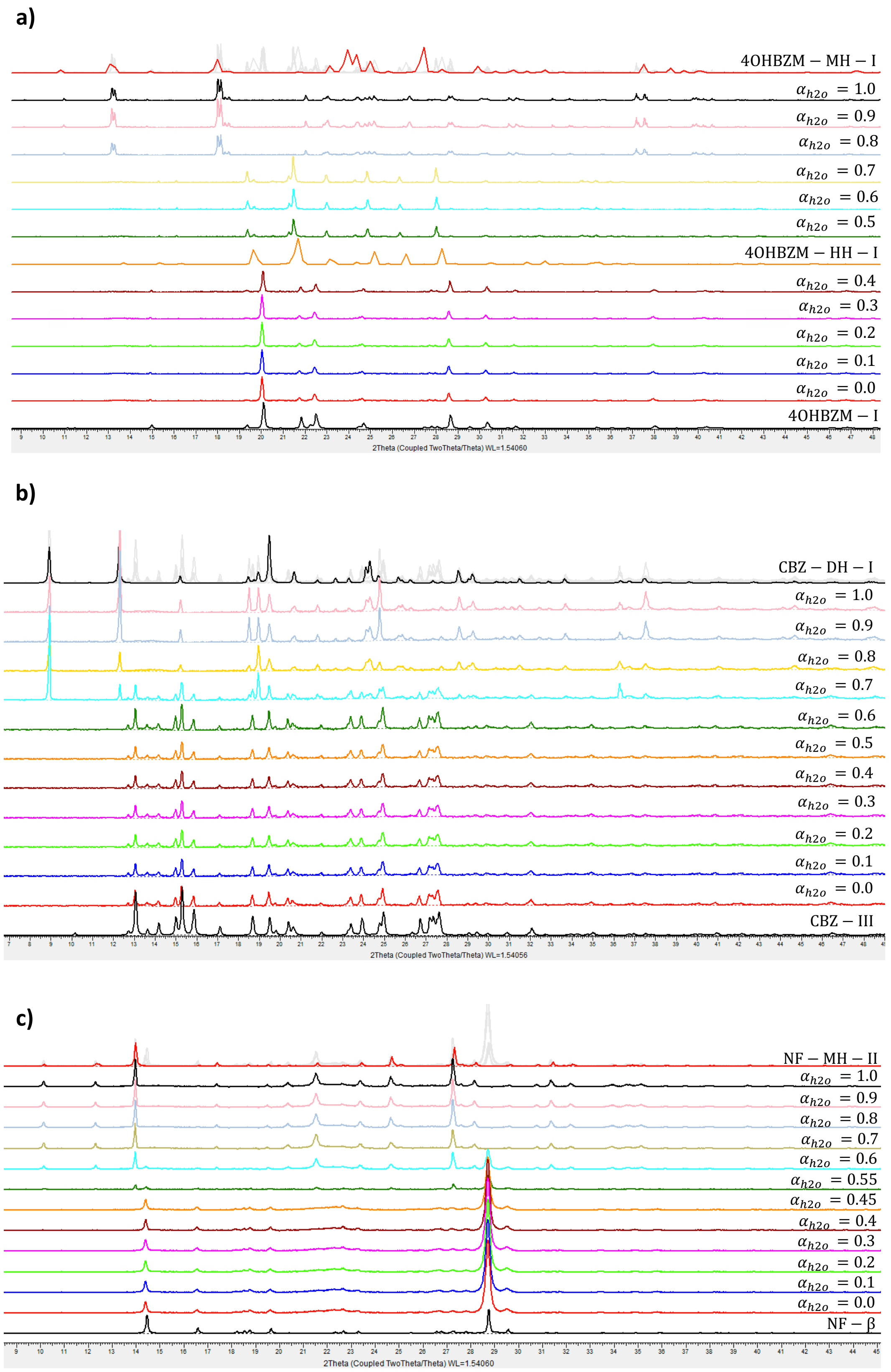

***Figure S 6**:* Anhydrous/hydrate forms obtained from *CSA-LAG* at 25±1°C and different water activities for a) 4ohbzm with **h_2o**:eg, b) cbz with **h_2o**:etoh and c) nf with **h_2o**:eg. Diffractograms for theo with **h_2o**:ace are given in Figure 4 in the main manuscript. Kapton background subtracted across all diffractograms.

Table S 12: Solid form outcomes from CSA-LAG experiments at 25±1°C for solvates exploration experiments for all four API systems. Active solvents are either dmso, ace or dmf. Carrier solvent used is always etoh.

API	CSA-LAG mixture **active:**carrier	$\alpha_{active\ solvent}$	Forms Identified
theo	**dmso**:etoh	0.000	THEO-II
theo	**dmso**:etoh	0.100	THEO-II
theo	**dmso**:etoh	0.200	THEO-II
theo	**dmso**:etoh	0.300	THEO-DMSO-I
theo	**dmso**:etoh	0.400	THEO-DMSO-I
theo	**dmso**:etoh	0.500	THEO-DMSO-I
theo	**dmso**:etoh	0.600	THEO-DMSO-I
theo	**dmso**:etoh	0.700	THEO-DMSO-I
theo	**dmso**:etoh	0.799	THEO-DMSO-I
theo	**dmso**:etoh	0.900	THEO-DMSO-I
theo	**dmso**:etoh	1.000	THEO-DMSO-I
4ohbzm	**ace**:eg	0.000	4OHBZM-I
4ohbzm	**ace**:eg	0.100	4OHBZM-I
4ohbzm	**ace**:eg	0.200	4OHBZM-I
4ohbzm	**ace**:eg	0.300	4OHBZM-I + 4OHBZM-ACET-I
4ohbzm	**ace**:eg	0.401	4OHBZM-I + 4OHBZM-ACET-I
4ohbzm	**ace**:eg	0.508	4OHBZM-I + 4OHBZM-ACET-I
4ohbzm	**ace**:eg	0.600	4OHBZM-I + 4OHBZM-ACET-I
4ohbzm	**ace**:eg	0.700	4OHBZM-I + 4OHBZM-ACET-I
4ohbzm	**ace**:eg	0.799	4OHBZM-ACET-I
4ohbzm	**ace**:eg	0.901	4OHBZM-ACET-I
4ohbzm	**ace**:eg	1.000	4OHBZM-ACET-I
cbz	**dmso**:etoh	0.000	CBZ-III
cbz	**dmso**:etoh	0.100	CBZ-III
cbz	**dmso**:etoh	0.200	CBZ-III
cbz	**dmso**:etoh	0.300	CBZ-DMSO-I + CBZ-III
cbz	**dmso**:etoh	0.400	CBZ-DMSO-I
cbz	**dmso**:etoh	0.500	CBZ-DMSO-I
cbz	**dmso**:etoh	0.600	CBZ-DMSO-I
cbz	**dmso**:etoh	0.700	CBZ-DMSO-I
cbz	**dmso**:etoh	0.799	CBZ-DMSO-I
cbz	**dmso**:etoh	0.900	CBZ-DMSO-I
cbz	**dmso**:etoh	1.000	CBZ-DMSO-I
nf	**dmf**:etoh	0.000	NF-β
nf	**dmf**:etoh	0.100	NF-DMF-II + NF-β
nf	**dmf**:etoh	0.200	NF-DMF-II
nf	**dmf**:etoh	0.301	NF-DMF-II
nf	**dmf**:etoh	0.401	NF-DMF-II
nf	**dmf**:etoh	0.500	NF-DMF-II
nf	**dmf**:etoh	0.601	NF-DMF-II
nf	**dmf**:etoh	0.701	NF-DMF-II
nf	**dmf**:etoh	0.802	NF-DMF-II
nf	**dmf**:etoh	0.900	NF-DMF-II
nf	**dmf**:etoh	1.000	NF-DMF-II

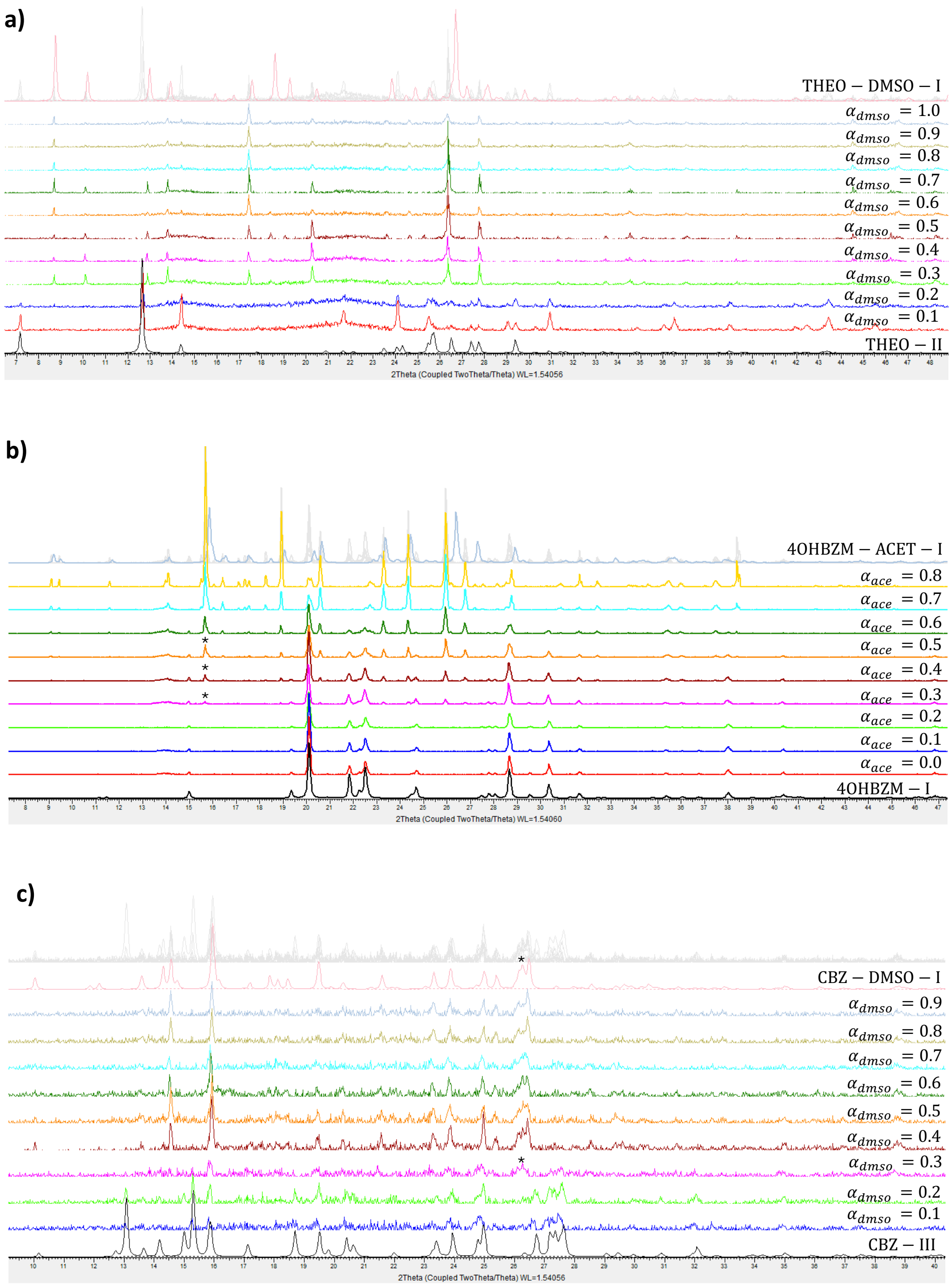

Figure S7: Anhydrous/solvated forms obtained from *CSA-LAG* at 25±1°C and different active solvent activities for a) theo with **dmso**:etoh , b) 4ohbzm with **ace**:eg and c) cbz with **dmso**:etoh. Diffractograms for the nf with **dmf**:etoh system are given in Figure 4 in the main manuscript. Kapton background subtracted across all diffractograms.

2.4 Effective temperature working window under CSA-LAG

To evaluate the potential impact of milling-induced heating during CSA-LAG the effective initial and final temperatures of experiments was monitored using an infrared thermometer (ETEKCITY Lasergrip 1080). Starting and final jar temperatures for two sample sets are presented, providing an estimate of the working temperature window during CSA-LAG. The corresponding data for theophylline anhydrate and nitrofurantoin anhydrate CSA-LAG experiments are summarised in Tables S13 and S14 respectively.

Table S 13: CSA-LAG experiments on theophylline anhydrous (THEO-II) using **dmso**:etoh liquid mixtures. Reported values include API mass, liquid volume and dsmo activity, along with initial and final milling temperatures.

**Composition** (200mg)	**Liquid mixture** (**dmso**:etoh) 200μL	**Activity** α_{dmso}	**Starting** T (°C)	**Final** T (°C)	**Jar position**
THEO-II	**dmso**:etoh	0.0	19.8	23.9	L
THEO-II	**dmso**:etoh	0.1	19.9	24.1	R
THEO-II	**dmso**:etoh	0.2	20.1	24.4	L
THEO-II	**dmso**:etoh	0.3	20.0	24.3	R
THEO-II	**dmso**:etoh	0.4	20.4	25.1	L
THEO-II	**dmso**:etoh	0.5	20.2	25.3	R
THEO-II	**dmso**:etoh	0.6	19.6	24.8	L
THEO-II	**dmso**:etoh	0.7	19.7	24.9	R
THEO-II	**dmso**:etoh	0.8	20.3	25.2	L
THEO-II	**dmso**:etoh	0.9	20.4	25.3	R
THEO-II	**dmso**:etoh	1.0	19.6	25.0	L
			Average 20.0	Average 24.8	

Table S 14: CSA-LAG experiments on nitrofurantoin anhydrous (NF-β) using **h2o**:eg liquid mixtures. Reported values include API mass, liquid volume and water activity, along with initial and final milling temperatures.

**Composition** (200mg)	**Liquid mixture** (**h2o**:eg) 200μL	**Activity** α_{h2o}	**Starting** T (°C)	**Final** T (°C)	**Jar position**
NF-β	**h2o**:eg	0.0	19.8	24.8	L
NF-β	**h2o**:eg	0.1	19.5	24.1	R
NF-β	**h2o**:eg	0.2	19.6	24.4	L
NF-β	**h2o**:eg	0.3	19.9	24.9	R
NF-β	**h2o**:eg	0.4	20.1	24.8	L
NF-β	**h2o**:eg	0.5	20.2	25.6	R
NF-β	**h2o**:eg	0.6	20.2	25.4	L
NF-β	**h2o**:eg	0.7	20.7	25.6	R
NF-β	**h2o**:eg	0.8	19.5	24.9	L
NF-β	**h2o**:eg	0.9	19.7	25.0	R
NF-β	**h2o**:eg	1.0	20.0	25.5	L
			Average 19.9	Average 25.1	

2.5 CSA-LAG Experiments of Competing Solvates with Binary Liquid Mixtures

Binary solvent mixtures of h2o:dmf were prepared to study the competing solvation of nf with h2o and dmf. Details on the mixtures, mol fractions mixture properties, and resulting forms listed below.

Table S 15: Forms of nf identified by CSA-LAG with h2o:dmf liquid mixtures. Mol fractions (x) and calculated solvent activities are given for all solvents.

x_{h2o}	x_{dmf}	α_{h2o}	α_{dmf}	$\alpha_{h2o}/\alpha_{dmf}$	$\alpha_{dmf}/\alpha_{h2o}$	Form(s) Identified
0.00	1.00	0.00	1.00	0.0	10000.0	NF-DMF-II
0.07	0.93	0.05	0.93	0.1	18.6	NF-DMF-II
0.15	0.85	0.11	0.85	0.1	8.0	NF-DMF-II
0.22	0.78	0.17	0.76	0.2	4.6	NF-DMF-II
0.31	0.69	0.24	0.66	0.4	2.7	NF-DMF-II
0.41	0.59	0.33	0.56	0.6	1.7	NF-DMF-II
0.52	0.48	0.45	0.43	1.0	1.0	NF-DMF-II
0.63	0.37	0.58	0.31	1.9	0.5	NF-DMF-II
0.70	0.30	0.66	0.24	2.8	0.4	NF-DMF-II
0.71	0.29	0.67	0.23	2.9	0.3	NF-MH-II
0.74	0.26	0.70	0.20	3.5	0.3	NF-MH-II
0.76	0.24	0.73	0.18	4.1	0.2	NF-MH-II
0.89	0.11	0.88	0.07	12.4	0.1	NF-MH-II
0.95	0.05	0.95	0.03	31.4	0.0	NF-MH-II
1.00	0.00	1.00	0.00	10000.0	0.0	NF-MH-II

2.6 CSA-LAG Experiments of Competing Solvates with Ternary Liquid Mixtures

Thermodynamic properties of the h2o:etoh:dmf liquid mixtures used in CSA-LAG with nf are presented in table S16 with corresponding molar fractions, solvent activities and fraction of activities.

Table S16: Forms of nf identified by CSA-LAG with h2o:etoh:dmf liquid mixtures. Mol fractions (x) and calculated solvent activities are given for all solvents.

Set	x_{h2o}	x_{etoh}	x_{dmf}	α_{h2o}	α_{etoh}	α_{dmf}	$\alpha_{h2o}/\alpha_{dmf}$	Form(s) Identified
1	0.79	0.12	0.09	0.82	0.26	0.11	7.8	NF-MH-II
2	0.73	0.15	0.11	0.79	0.32	0.06	14.2	NF-MH-II
3	0.65	0.20	0.15	0.72	0.35	0.07	9.8	NF-MH-II
4	0.48	0.30	0.22	0.59	0.40	0.12	5.1	NF-MH-II
5	0.16	0.48	0.36	0.26	0.44	0.23	1.1	NF-DMF-II
6	0.08	0.52	0.39	0.15	0.45	0.26	0.6	NF-DMF-II
7	0.04	0.55	0.41	0.08	0.45	0.28	0.3	NF-DMF-II
8	0.09	0.06	0.85	0.07	0.03	0.84	0.1	NF-DMF-II
9	0.07	0.91	0.02	0.18	0.92	0.01	21.9	NF-β
10	0.70	0.22	0.08	0.78	0.41	0.03	22.5	NF-MH-II
11	0.60	0.19	0.21	0.67	0.30	0.12	5.8	NF-MH-II
12	0.50	0.15	0.35	0.53	0.20	0.24	2.2	NF-DMF-II
13	0.37	0.11	0.52	0.36	0.11	0.43	0.8	NF-DMF-II
14	0.28	0.52	0.20	0.46	0.58	0.09	5.1	NF-β
15	0.23	0.44	0.33	0.36	0.44	0.19	1.9	NF-DMF-II
16	0.20	0.38	0.42	0.28	0.34	0.29	1.0	NF-DMF-II
17	0.17	0.32	0.51	0.21	0.25	0.40	0.5	NF-DMF-II
18	0.15	0.27	0.58	0.17	0.20	0.48	0.4	NF-DMF-II
19	0.14	0.66	0.20	0.30	0.66	0.09	3.3	NF-β
20	0.13	0.58	0.29	0.24	0.55	0.16	1.5	NF-DMF-II
21	0.07	0.62	0.31	0.14	0.56	0.18	0.8	NF-DMF-II
22	0.08	0.74	0.18	0.20	0.72	0.08	2.4	NF-β
23	0.44	0.41	0.15	0.60	0.52	0.07	9.0	NF-MH-II
24	0.34	0.31	0.35	0.43	0.33	0.22	1.9	NF-DMF-II
25	0.30	0.28	0.42	0.36	0.27	0.29	1.2	NF-DMF-II
26	0.27	0.25	0.48	0.32	0.23	0.36	0.9	NF-DMF-II
27	0.03	0.65	0.32	0.06	0.57	0.20	0.3	NF-DMF-II
28	0.03	0.70	0.26	0.08	0.65	0.14	0.5	NF-DMF-II
29	0.03	0.80	0.16	0.11	0.78	0.07	1.5	NF-β
30	0.42	0.13	0.44	0.43	0.14	0.35	1.2	NF-DMF-II
31	0.60	0.09	0.31	0.60	0.14	0.22	2.7	NF-DMF-II & NF-MH-II
32	0.66	0.10	0.23	0.68	0.19	0.15	4.7	NF-MH-II
33	0.04	0.86	0.11	0.12	0.85	0.04	2.9	NF-β
34	0.55	0.35	0.10	0.69	0.51	0.04	17.0	NF-MH-II
35	0.40	0.50	0.10	0.60	0.61	0.04	14.9	NF-MH-II
36	0.30	0.60	0.10	0.51	0.67	0.04	12.8	NF-β
37	0.20	0.70	0.10	0.40	0.74	0.04	10.1	NF-β

2.7 Investigating Critical Water Activity Transferability

The critical water activity at a given temperature and pressure should host true regardless of different solvent mixtures used to identify the boundaries. This is further supported by the thermodynamic laws and equations governing hydration. In an effort to evaluate transferability and capabilities of CSA-LAG, they values obtained through experimentation were compared against different solvent systems. These data are highlighted in tables S17 and S18 for two model systems nitrofurantoin and theophylline respectively. Critical activities for the same active solvent remain identical independently of the carrier solvent used and the method of achieving equilibrium (CSA-LAG or slurry). This further emphasises the need to report values in terms of activities and not compositions.

Table S17: Comparison of observed hydrate/anhydrate phase boundaries at ~25C° for nitrofurantoin, comparing different solvent mixtures used and both CSA-LAG and slurring.

Equilibrium reaction	Active solvent	Passive solvent	Method Used	Critical water activity	Water mol fraction range	Source
NF-β + H_2O ⇌ NF-MH-I	water	Ethylene glycol	slurring	0.45-0.55	0.478-0.571	This work
NF-β + H_2O ⇌ NF-MH-I	water	Ethylene glycol	CSA-LAG	0.45-0.55	0.478-0.571	This work
NF-β + H_2O ⇌ NF-MH-I	water	acetone	slurring	0.43-0.56	0.094-0.149	Asproudis et al., 2022[23]
NF-β + H_2O ⇌ NF-MH-I	water	acetone	CSA-LAG	0.45-0.5	0.101-0.121	This work
NF-β + H_2O ⇌ NF-MH-I	water	ethanol	CSA-LAG	0.445-0.51	0.2-0.2637	This work

Table S18: Comparison of observed hydrate/anhydrate phase boundaries at ~25C° for theophylline, comparing different solvent mixtures used and both CSA-LAG and slurring.

Equilibrium reaction	Active solvent	Passive solvent	Method Used	Critical water activity	Water mol fraction range	Source
THEO-II + H_2O ⇌ THEO-MH-I	water	methanol	slurring	0.56-0.61	0.525-0.579	Ticehurst et al., 2002[24]
THEO-II + H_2O ⇌ THEO-MH-I	water	acetone	CSA-LAG	0.5-0.6	0.121-0.175	This work
THEO-II + H_2O ⇌ THEO-MH-I	water	Ethylene glycol	CSA-LAG	0.5-0.6	0.521-0.618	This work

2.8 Crystal structures determined by SCXRD

Over the course of this work, two new solvates were identified, namely 4OHBZM-ACET-I and NF-DMF-II. The resolved structures for both solvates were deposited in the CSD and have deposition numbers 2443101 and 443102. A summary of the crystal and refinement data is given in Table S17.

Table S 19: Crystal and refinement data for 4OHBZM-ACET-I and NF-DMF-II.

	**4OHBZM-ACET-I**	**NF-DMF-II**
CCDC Deposition number	2443101	2443102
Empirical formula	$C_{17}H_{20}N_2O_5$	$C_{11}H_{13}N_5O_6$
Formula weight	332.359	311.26
Temperature/K	120.00	100.00
Crystal system	triclinic	triclinic
Space group	P-1	P-1
a/Å	7.8263(3)	6.06790(10)
b/Å	10.7085(4)	6.59890(10)
c/Å	11.5341(5)	17.4698(4)
α/°	115.471(1)	98.248(2)
β/°	103.558(1)	93.825(2)
γ/°	90.429(1)	100.8570(10)
Volume/Å^3	841.89(6)	676.72(2)
Z	2	2
ρ_{calc}g/cm^3	1.311	1.528
μ/mm^{-1}	0.097	0.118
F(000)	352.3	324.0
Crystal size/mm^3	0.289 × 0.247 × 0.179	0.108 × 0.011 × 0.01
Radiation	Mo Kα (λ = 0.71073)	Synchrotron (λ = 0.68890)
2Θ range for data collection/°	4.06 to 65.36	2.294 to 64.272
Index ranges	-11 ≤ h ≤ 11, -16 ≤ k ≤ 16, -17 ≤ l ≤ 17	-9 ≤ h ≤ 9, -10 ≤ k ≤ 10, -26 ≤ l ≤ 25
Reflections collected	25605	12836
Independent reflections	6159 [R_{int} = 0.0275, R_{sigma} = 0.0239]	4483 [R_{int} = 0.0371, R_{sigma} = 0.0305]
Data/restraints/parameters	6159/0/243	4483/0/205
Goodness-of-fit on F^2	1.055	1.105
Final R indexes [I>=2σ (I)]	R_1 = 0.0393, wR_2 = 0.1105	R_1 = 0.0447, wR_2 = 0.1272
Final R indexes [all data]	R_1 = 0.0463, wR_2 = 0.1163	R_1 = 0.0491, wR_2 = 0.1310
Largest diff. peak/hole / e Å^{-3}	0.43/-0.23	0.56/-0.30

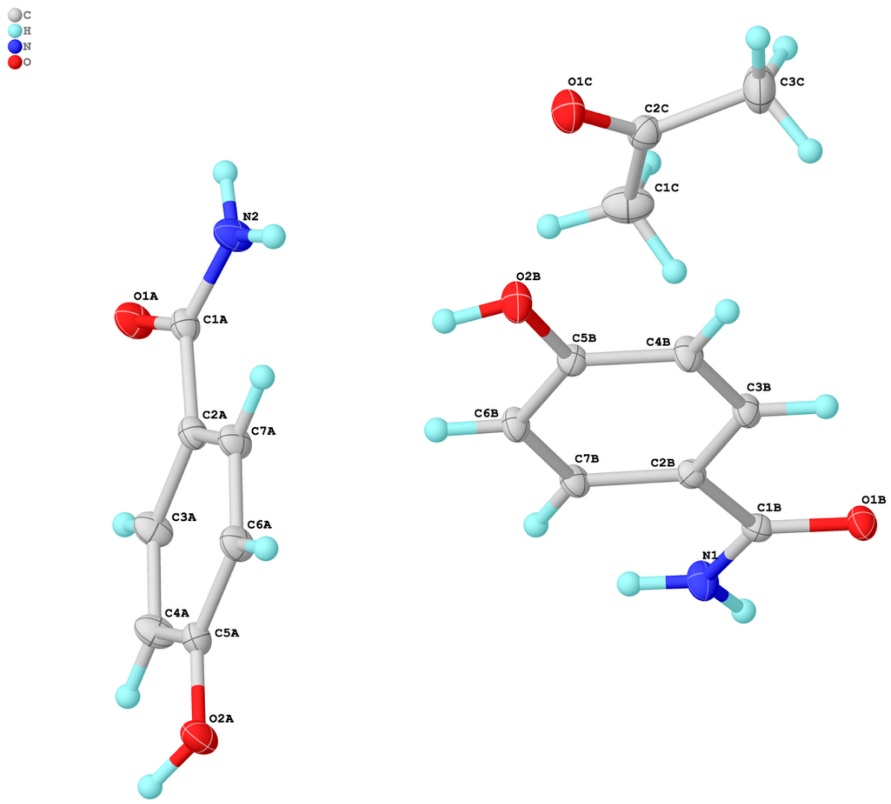

***Figure S8**:* Asymmetric units of the resolved structure 2443101 4OHBZM-ACET-I.

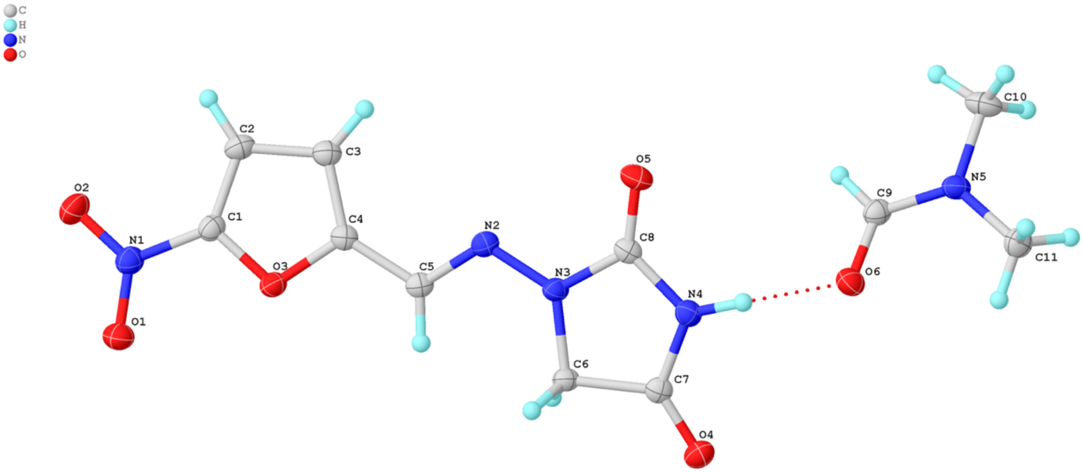

***Figure S9**:* Asymmetric units of the resolved structure 2443102 NF-DMF-II.

Supplementary References

1. Aspen Technology Inc. Aspen Plus, V14. Preprint at (2024).

2. Hilfiker, Rolf; Raumer von, M. *Polymorphism in the Pharmaceutical Industry: Solid Form and Drug Development*. *Wiley.com* (2019).

3. Vetere, A. The NRTL equation as a predictive tool for vapor-liquid equilibria. *Fluid Phase Equilib* **218**, 33–39 (2004).

4. Elliott, J. Richard. & Lira, C. T. *Introductory Chemical Engineering Thermodynamics*. (Prentice Hall, 2011).

5. Reutzel-Edens, S. M., Braun, D. E. & Newman, A. W. Hygroscopicity and Hydrates in Pharmaceutical Solids. in *Polymorphism in the Pharmaceutical Industry* 159–188 (Wiley, 2018).

6. Firaha, D. *et al.* Predicting crystal form stability under real-world conditions. *Nature* **623**, 324–328 (2023).

7. Zhu, H. & Grant, D. J. W. Influence of water activity in organic solvent + water mixtures on the nature of the crystallizing drug phase. 2. Ampicillin. *Int J Pharm* **139**, 33–43 (1996).

8. Zhu, H., Yuen, C. & Grant, D. Influence of water activity in organic solvent + water mixtures on the nature of the crystallizing drug phase. 1. Theophylline. *Int J Pharm* **135**, 151–160 (1996).

9. D'Abbrunzo, I. *et al.* Competitive Mechanochemical Solvate Formation of Theophylline in the Presence of Miscible Liquid Mixtures. *Cryst Growth Des* **23**, 8094–8102 (2023).

10. Khankari, R. K. & Grant, D. J. W. Pharmaceutical hydrates. *Thermochim Acta* **248**, 61–79 (1995).

11. Davey, R. J. & Cardew, P. T. The kinetics of solvent-mediated phase transformations. *Proceedings of the Royal Society of London. A. Mathematical and Physical Sciences* **398**, 415–428 (1985).

12. Basford, P. A. *et al.* Impact of Crystal Structure and Molecular Conformation on the Hydration Kinetics of Fluconazole. *Cryst Growth Des* **19**, 7193–7205 (2019).

13. Evans, P. Scaling and assessment of data quality. *Acta Crystallogr D Biol Crystallogr* **62**, 72–82 (2006).

14. Evans, P. R. & Murshudov, G. N. How good are my data and what is the resolution? *Acta Crystallogr D Biol Crystallogr* **69**, 1204–1214 (2013).

15. Winn, M. D. *et al.* Overview of the *CCP* 4 suite and current developments. *Acta Crystallogr D Biol Crystallogr* **67**, 235–242 (2011).

16. Winter, G. xia2: an expert system for macromolecular crystallography data reduction. *J Appl Crystallogr* **43**, 186–190 (2010).

17. Winter, G. *et al.* DIALS: implementation and evaluation of a new integration package. *Acta Crystallogr D Struct Biol* **74**, 85–97 (2018).

18. Dolomanov, O. V., Bourhis, L. J., Gildea, R. J., Howard, J. A. K. & Puschmann, H. OLEX2: a complete structure solution, refinement and analysis program. *J Appl Crystallogr* **42**, 339–341 (2009).

19. Sheldrick, G. M. SHELXT – Integrated space-group and crystal-structure determination. *Acta Crystallogr A Found Adv* **71**, 3–8 (2015).

20. Sheldrick, G. M. Crystal structure refinement with SHELXL. *Acta Crystallogr C Struct Chem* **71**, 3–8 (2015).

21. Coelho, A. A., Evans, J., Evans, I., Kern, A. & Parsons, S. The TOPAS symbolic computation system. *Powder Diffr* **26**, S22–S25 (2011).

22. Coelho, A. A. TOPAS and TOPAS-Academic: an optimization program integrating computer algebra and crystallographic objects written in C++. *J Appl Crystallogr* **51**, 210–218 (2018).

23. Asproudis, I. Losing water – towards understanding the dehydration kinetics of pharmaceutical hydrates. (The University of Edinburgh, Edinburgh UK, 2022).

24. Ticehurst, M. D., Storey, R. A. & Watt, C. Application of slurry bridging experiments at controlled water activities to predict the solid-state conversion between anhydrous and hydrated forms using theophylline as a model drug. *Int J Pharm* **247**, 1–10 (2002).
